# Quantification of edematous changes by diffusion magnetic resonance imaging in gastrocnemius muscles after spinal nerve ligation

**DOI:** 10.1371/journal.pone.0193306

**Published:** 2018-02-22

**Authors:** Koji Abe, Toshiyasu Nakamura, Eiko Yamabe, Koichi Oshio, Takeshi Miyamoto, Masaya Nakamura, Morio Matsumoto, Kazuki Sato

**Affiliations:** 1 Department of Orthopaedic Surgery, School of Medicine, Keio University, Tokyo, Japan; 2 Clinical Research Center, International University of Health and Welfare, Tokyo, Japan; 3 Department of Orthopaedic Surgery, Saiseikai Yokohama-shi Tobu Hospital, Kanagawa, Japan; 4 Department of Diagnostic Radiology, School of Medicine, Keio University, Tokyo, Japan; University of PECS Medical School, HUNGARY

## Abstract

Patients with complex regional pain syndrome (CRPS) exhibit diverse symptoms, such as neuropathic pain, allodynia, local edema and skin color changes in the affected lesion. Although nerve injury may cause CRPS, pathophysiological mechanisms underlying the syndrome are unclear, and local edema, a characteristic of CRPS, has not been evaluated quantitatively for technical reasons. Here, using a rat spinal nerve ligation-induced CRPS model, we show that edematous changes in gastrocnemius muscle can be detected quantitatively by diffusion magnetic resonance imaging (MRI). Using the line-scan diffusion spectrum on a 1.5 T clinical MR imager, we demonstrate significant elevation of the apparent diffusion coefficient (ADC) ratios in gastrocnemius muscle on the ligated versus the sham-operated rats by one day after surgery, those ratios gradually decreased over time. Meanwhile, T2 ratios in gastrocnemius muscle on the ligated rats increased gradually and significantly, peaking two weeks after surgery, and those ratios remained high and were consistent with edema. Expression of vascular endothelial growth factor (VEGF), a key regulator of blood vessel formation and function, was significantly lower in gastrocnemius muscle on the ligated versus non-ligated side, suggesting that nerve ligation promotes edematous changes and perturbs VEGF expression in target muscle.

## Introduction

Complex regional pain syndrome (CRPS) is a neuropathic disorder characterized by continuing regional pain, which is incompatible with the usual course of general trauma or other lesions with respect to the time course or degree. CRPS shows variable progression over time [1–3]. Affected patients exhibit local edematous changes suggestive of autonomic nervous system dysfunction, such as altered sweating, skin color, and skin temperature. Nonetheless, mechanisms underlying these disorders are unclear, and multiple factors, among them peripheral and central sensitization, inflammation, altered sympathetic and catecholaminergic function, altered somatosensory representation in the brain, genetic factors, and psychophysiological interactions, likely play a role in CRPS development [4]. Our lack of knowledge of the underlying pathophysiology of CRPS has hampered efforts to develop definitive treatment, although existing approaches are reportedly more effective when started at an early stage [5]. Moreover, it is currently difficult to objectively diagnose or evaluate treatment efficacy in CRPS.

Nonetheless, objective diagnostic tests for CRPS have been proposed, such as plain radiographs to detect regional osteopenia [6, 7], functional MRI to detect patients’ brain responses [8], bone scintigraphy to assess regional bone loss [9–12], digital temperature measurements [13–15], laser Doppler fluxmetry [16] and vital capillaroscopy to analyze digital blood flow [17]. In clinical settings, diagnostic criteria for CRPS defined by the International Association for the Study of Pain (IASP) [1] have been widely used, and recently, diagnostic Budapest Criteria [2] have become popular. In both, edema is defined as one of the most important CRPS symptoms [1–3], one that could also serve to assess CRPS treatment effects.

Tumor Necrosis Factor α (TNFα) is a well-characterized inflammatory cytokine that promotes regional edematous changes [18, 19]. Moreover, vascular endothelial growth factor (VEGF) is required for endothelial cell formation and function, and excessive or deficient VEGF levels promote leaky blood vessel formation or dysfunction, respectively [20–22]. To date, changes in cytokine levels are implicated in CRPS development. Expression of inflammatory cytokines such as TNFα, bradykinin, Substance P, calcitonin gene-related peptide (cGRP) and IL-10 reportedly is deregulated in blood, skin, blister fluid or cerebrospinal fluid of CRPS patients [4, 23]. Similarly, in animal models of CRPS, elevated levels of Substance P, cGRP, TNFα or VEGF are detected in blood, skin and injured nerves [4] [23] [24].

Magnetic Resonance imaging (MRI) is a non-invasive diagnostic imaging device whose output is totally independent of examiner bias. Diffusion MRI is a form of MRI based on measuring random Brownian motion of water molecules. Both apparent diffusion coefficient (ADC) and T2 values reflect the state of extracellular fluid [25–28]. Previously, a T2-high value accompanied by high ADC as detected by diffusion MRI has been correlated with edema in brain and heart [26, 29–34]. However, detection of edematous changes in CRPS by assessing T2 combined with ADC values using diffusion MRI has not been reported.

Here, we sequentially measured ADC and T2 values in skeletal muscle of the lower extremity using diffusion MRI and detected early elevation of both, indicative of edematous changes in rat models. We also analyzed changes in cytokine expression in edematous muscle in those models and observed down-regulation of *VEGF*.

## Materials and methods

### Experimental model

Ten 9-week-old female Wistar rats, each weighing approximately 200g, were used. The rats were housed in standard plastic cages enriched with pulp chips bedding under specific pathogen-free conditions. They were kept under constant environmental conditions with 12-hour light-dark cycle at a temperature of 23 °C and humidity of 55%. Sterile water and sterile irradiated chow were available *ad libitum*. All efforts were made to minimize suffering. The study was approved by our university institutional animal use and care committee (09030). Rats were anesthetized with 40 mg/kg pentobarbital (Somnopentyl; Kyoritsu Seiyaku Corp., Chiyoda-ku, Tokyo, Japan) injected intraperitoneally. Spinal nerve ligation (SNL) was performed as described by Kim and Chung [35, 36]. Under a dissecting microscope, we removed the left L6 transverse process and ligated the ipsilateral L5 spinal nerve tightly with 6–0 silk thread in 5 rats. Sham surgery including nerve dissection only was performed as a control in the remaining 5 rats. No analgesics were administered post-operatively to eliminate the influence on mechanical sensitivity. After surgery, the rats were housed under the same conditions and monitored daily for the first 7 days and wekkly for the following 5 weeks for the wound healing and signs of abnormal physical and behavioral conditions. The methods of euthanasia consisted of an overdose of sodium pentobarbital injected intraperitoneally (> 100mg/kg).

### Behavioral testing: Mechanical allodynia

To quantify mechanical sensitivity, foot withdrawal in response to mechanical stimuli was measured using a Dynamic Plantar Aesthiometer (DPA, Ugo Basile, Milan, Italy) [37–43]. The DPA is an automated version of the von Frey hair assessment, in which mechanical stimuli are applied to the plantar surface of the foot using filaments, and the force at which the paw withdrawal occurred is recorded. Nirogi et al showed that they were able to assess mechanical allodynia of SNL model with DPA as accurately as with von Frey monofilaments. [42] Each paw was tested four times per session, and we analyzed mean values. We then calculated the ratio of the applied force on the operated limb to that on the contralateral control limb and defined it as the withdrawal ratio.

### MRI

An anesthetized rat was positioned on a custom fixator with its fore- and hind-feet held in the prone position. Starting at one, three, and five days after surgery, and then weekly for six more weeks, MR images of gastrocnemius muscle were obtained with a 1.5T clinical MR imager (Signa Excite HD, version 12: GE Medical Systems, Milwaukee, WI, USA). To do so, rats were set on a round surface coil 3 inches wide used clinically to image the human wrist. Transverse T2-weighted fast spin echo MR images were obtained. Sequence parameters were as follows: TR = 4000msec, TE = 77msec, matrix size = 128x128, and column thickness = 5mm. Acquired images were transferred to a personal computer and analyzed with a DICOM viewer (OsiriX) [44, 45].

### ADC and T2 values

**Fig 1:**
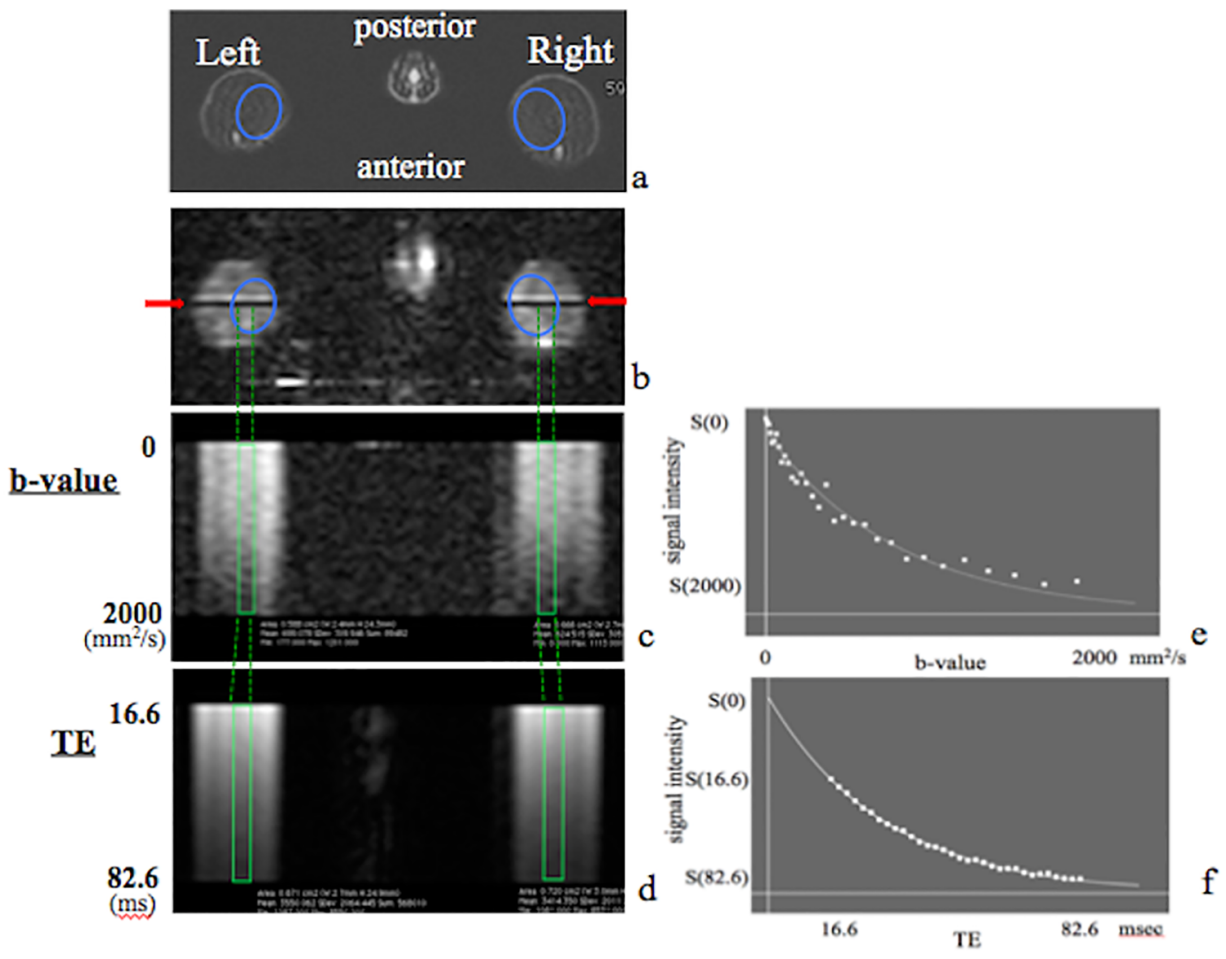
MRI and calculation of ADC and T2 values. a: Transverse T2-weighted MR image (TR/TE = 4000msec/77msec) of rat gastrocnemius muscle, outlined in ovals, when animal is in a prone position. b-d: A line (red arrows in (b)) is set across gastrocnemius muscle, outlined in ovals. A line scan diffusion spectrum was obtained to calculate the ADC value, in which the b-value is stepped from 0 to 2000 mm^2^/seconds in 32 steps (c). That approach was also used to calculate T2, in which TE was stepped from 16.6 to 82.6 msec in 32 steps (d). The area inside rectangles represents signals from gastrocnemius muscle. e,f: Signal intensities obtained from line-scan diffusion MRS data are plotted as an exponential curve. ADC and T2 values are calculated from the signal decay curve.

From a set of 5-mm-thick transverse T2-weighted images of the lower extremity, a single slice including gastrocnemius muscle was selected at calf level (Fig 1a). A line extending from left to right and passing through skin, subcutaneous fat, muscle was then chosen for interrogation in T1-weighted image (TR/TE = 1000msec/77msec) (Fig 1b). To measure the ADC, the signal decay of gastrocnemius muscle with an increasing b-value stepped from 0 to 2000 sec/mm^2^ in 32 steps was measured using a line-scan Stejskal-Tanner spin echo diffusion approach (TR/TE = 4000 msec/77 msec, matrix size = 128x128, thickness = 5.0mm) (Fig 1c) [46]. The motion probing gradients (MPG) for diffusion weighting were applied on all three axes simultaneously. Since the anisotropy is not significant at this location and signal-to-noise ratio (SNR), we chose to maximize the b-value for given TE. For diffusion imaging with b-values below 2000 sec/mm^2^, the standard model of monoexponential signal decay was occasionally appropriate [47], in which case the ADC was determined according to the following formula: S(b) = S(0)·exp(-b·D), where S is signal intensity, b is the b-value, and D is the ADC. To measure T2, the signal decay of the gastrocnemius muscle with TE stepped from 16.6 to 82.6 msec in 32 steps was fitted in the spin echo line-scan diffusion spectrum (TR = 4000msec, matrix size = 128x128, thickness = 5.0mm) (Fig 1d). T2 values were calculated by fitting the data to the following equation: S(TE) = S(0)·exp(-TE/T2), where S is signal intensity and TE is time to echo. From these formulas, the ADC is defined as a reciprocal of the time constant in the exponential curve, such as T2 value [45].

ADC and T2 values were calculated by fitting data to an exponential curve using custom calculating software (Fig 1e and 1f). We then calculated the ratio of ADC or T2 values of muscles on the ligated side to values obtained from the contralateral intact muscle in the same rat, to obtain an ADC ratio and a T2 ratio for each animal in both experimental and sham surgery control rats [45].

### Realtime PCR analysis

Total RNA was collected from gastrocnemius muscles using TRI Reagent (Molecular Research Center, Inc., Cincinnati, OH), and single-stranded complementary DNAs (cDNAs) were synthesized with reverse transcriptase (Clontech). Realtime PCR was performed using SYBR Premix ExTag II (Takara Bio Inc., Otsu, Shiga, Japan) and a DICE thermal cycler (Takara Bio Inc.), according to the manufacturer’s instructions. *β*-actin expression served as an internal control for realtime PCR, as described [48]. Primers used for this study are as below.

*β-actin*-forward: 5’-CCTAAGGCCAACCGTGAAAAG-3’

*β-actin*-reverse: 5’-GTCCATCACAATGCCAGTGGT-3’

*TNFα*-forward: 5’-TGTCTGTGCCTCAGCCTCTTC-3’

*TNFα*-reverse: 5’-CTGATGAGAGGGAGCCCATTT-3’

*VEGF*-forward: 5’-CTGGACCCTGGCTTTACTGCT-3’

*VEGF*-reverse: 5’-CAATAGCTGCGCTGGTAGACG-3’

### Statistical analysis

All data are presented as mean values ± standard error of the mean (SEM). Changes in ADC or T2 ratios on the operated versus contralateral side were compared statistically using one-way analysis of variance (ANOVA) with a Tukey post hoc test. Comparison between the nerve ligation group and control group was made using an unpaired t-test. Realtime PCR data are presented as mean *TNFα or VEGF expression* relative to *β-actin* ± SEM, and significance of differences between ligated and sham-operated groups was evaluated using Student's *t*-test. A *P* value <0.05 was considered significant.

## Results

### Rats subjected to spinal nerve ligation develop mechanical allodynia

**Fig 2:**
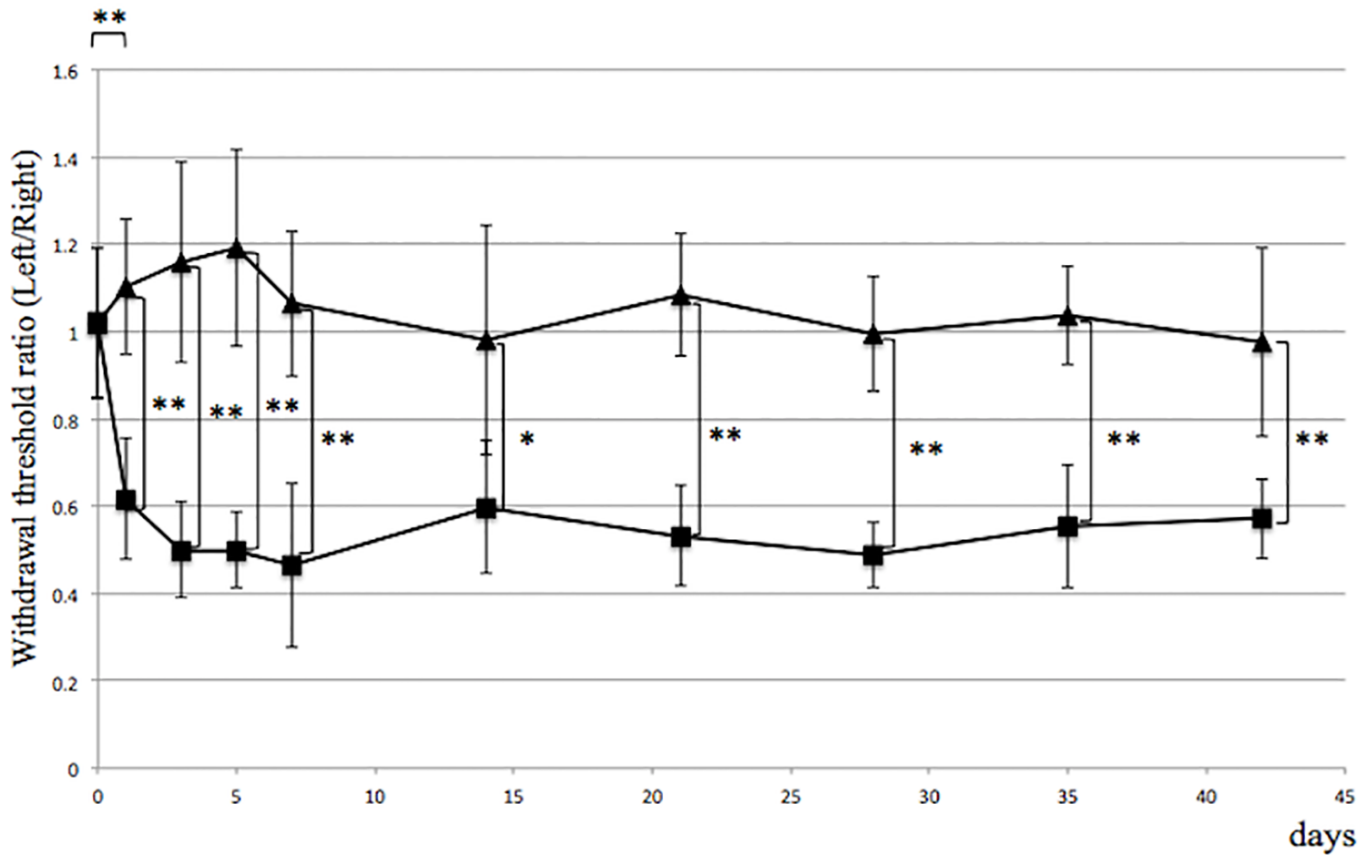
Mechanical allodynia develops following spinal nerve ligation. L5 spinal nerve ligation (ligation group) or sham surgery (control group) was performed on the left side in rats, and mechanical allodynia was compared with the non-operated right side. Allodynia was evident by one day after surgery in the ligation (■) compared to the control (▲) group. *p<0.05, **p<0.01, n = 5, error bars = SEM.

We performed left L5 spinal nerve ligation (ligation group) or sham surgery (control group) in nine-week-old female rats and monitored them for development of mechanical allodynia, a characteristic feature of CRPS. Animals were evaluated at indicated time points (Fig 2) after surgery by foot withdrawal in response to mechanical stimuli, as evaluated using the von Frey hair assessment test. In the ligation group, mechanical allodynia was evident one day after surgery with a withdrawal ratio of 0.615 ± 0.069, and persisted throughout the experimental period. In the control sham surgery group, we did not observe mechanical allodynia throughout the experimental period. Differences between groups in terms of withdrawal ratios were statistically significant throughout the study period, except for those assessed before surgery (Fig 2).

### ADC and T2 ratios increase in gastrocnemius muscle following spinal nerve ligation

**Fig 3:**
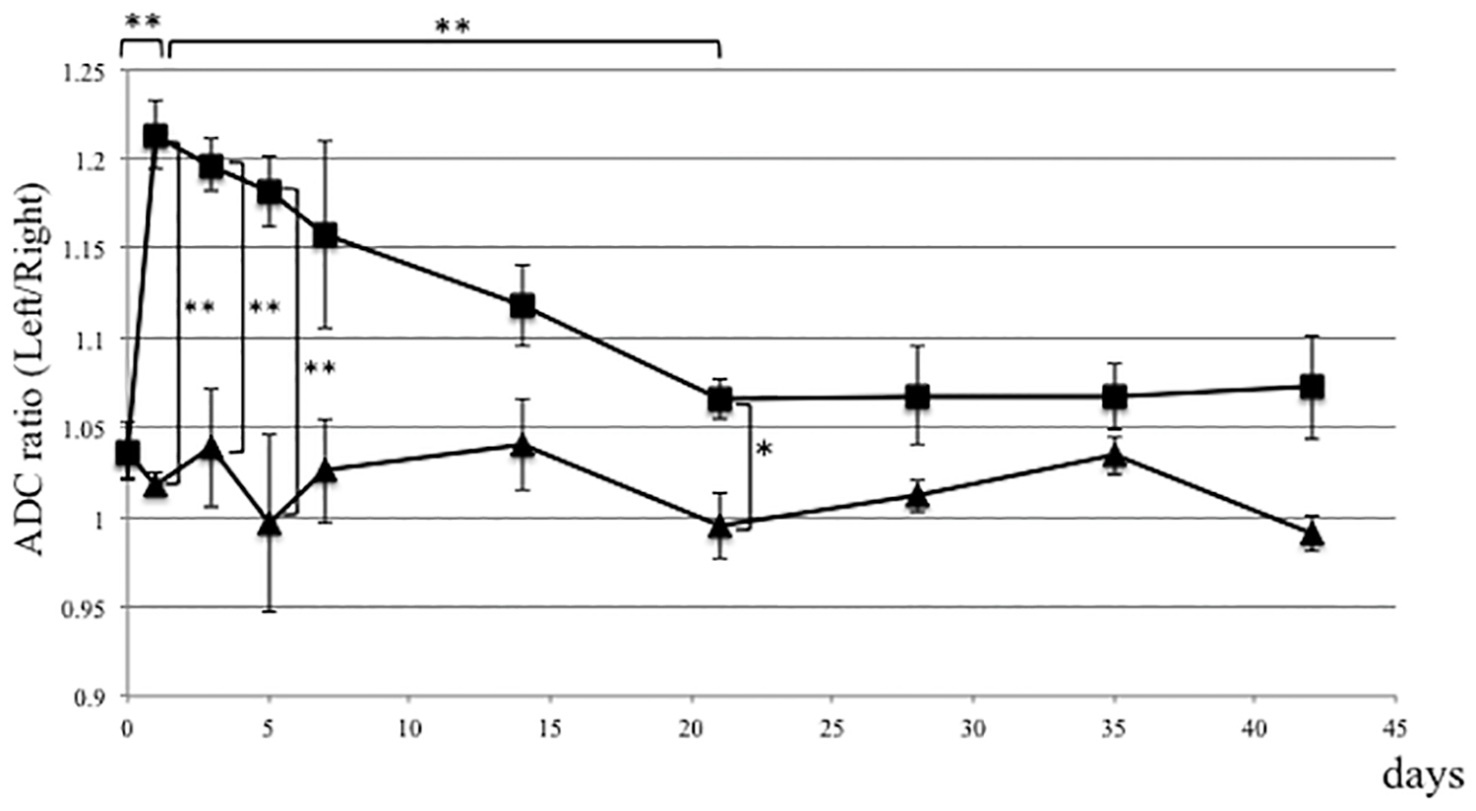
The ADC ratio is elevated in gastrocnemius muscle in the CRPS model. L5 spinal nerve ligation (ligation group) or sham surgery (control group) was performed on the left side of rats, and the ratio of ADC values in gastrocnemius muscle on the operated-side versus the contralateral side, as evaluated by diffusion MRI, was elevated in experimental relative to sham-operated controls at indicated time points after surgery. The ADC ratio increased rapidly by one day after nerve ligation and then gradually decreased. Differences between the nerve ligation (■) and sham surgery control (▲) groups were statistically significant at one, three, and five days post-surgery. *p<0.05, **p<0.01, n = 5, error bars = SEM.

**Fig 4:**
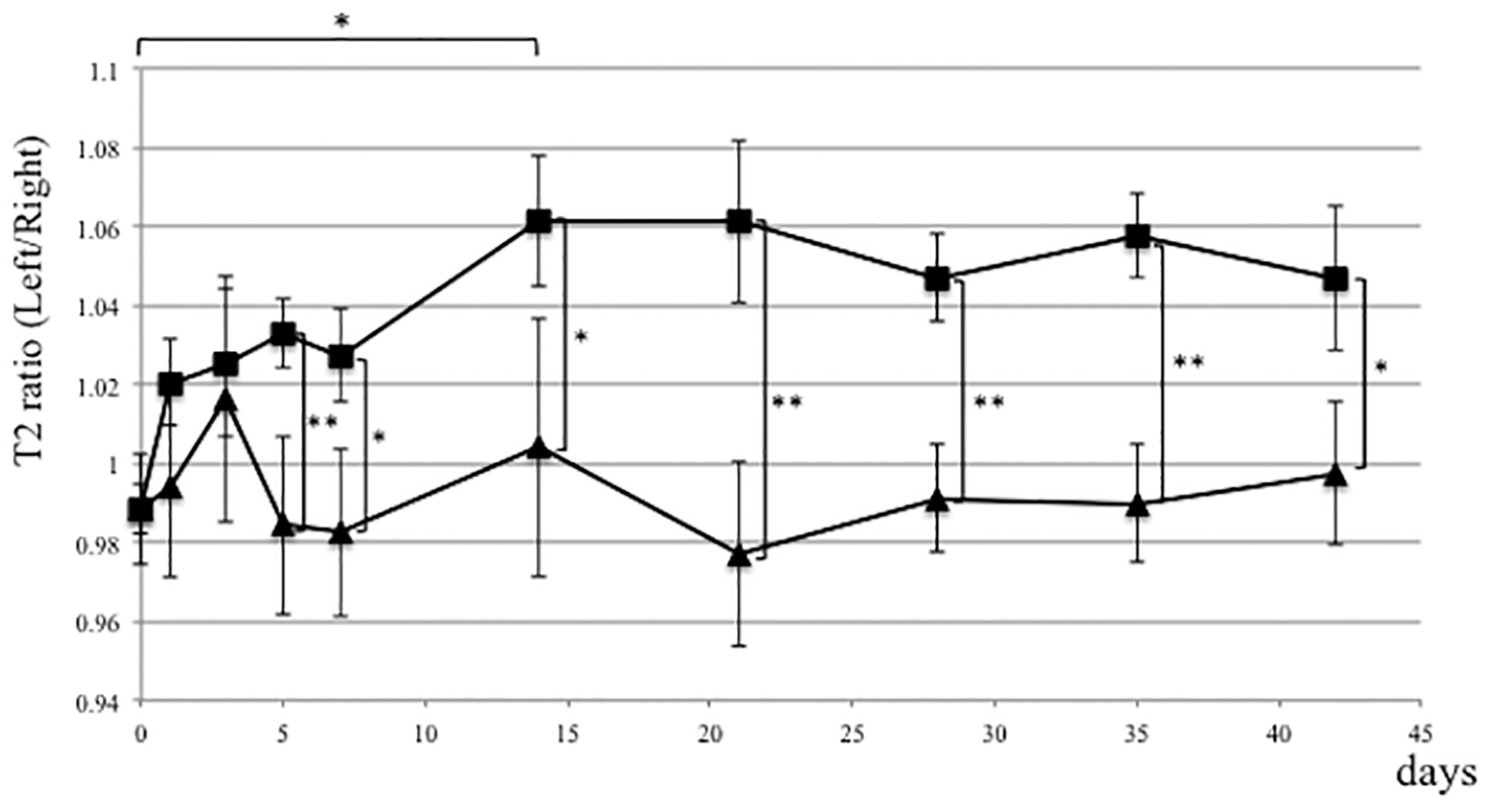
T2 ratio in gastrocnemius muscle is elevated in the CRPS model. L5 spinal nerve ligation (ligation group) or sham surgery (control group) was performed on the left side in rats, and the T2 ratio was evaluated by diffusion MRI at indicated time points after surgery. In the nerve ligation group, the T2 ratio of gastrocnemius muscle on the operated-side versus the contralateral side increased gradually after surgery. Differences in ratios between ligation (■) and sham surgery control (▲) groups were statistically significant by five days post-surgery. *p<0.05, **p<0.01, n = 5, error bars = SEM.

Concomitant elevation of ADC and T2 values reportedly indicate edematous changes in tissues [26, 29–34]. Here, we calculated these ratios by dividing ADC or T2 values of muscles on the ligated side by ADC or T2 values obtained from contralateral intact muscle of the same rat. These ratios were calculated both for experimental (ligation) and control (sham surgery) rats [45] (Fig 1). In the nerve ligation group, the ADC ratio of target gastrocnemius muscle increased rapidly immediately after ligation of the L5 nerve and was 1.213 ± 0.037 by day one, and then gradually decreased. ADC ratios in gastrocnemius muscle in the ligation group were significantly high compared to the control group at one, three, and five days post-surgery (*P* < 0.05) (Fig 3). In the control group, ADC ratios in gastrocnemius muscle of the sham-operated side were identical to those of contralateral intact muscle (Fig 3). Meanwhile, T2 ratios of gastrocnemius muscle in the ligation group increased gradually, and were significantly high compared to the control group by five days post-surgery (*P* < 0.05), and significant differences were observed throughout the experimental period (Fig 4). T2 ratios in gastrocnemius muscle of the control group were also identical to values on the contralateral side (Fig 4).

### VEGF expression decreases in gastrocnemius muscle following spinal nerve ligation

**Fig 5:**
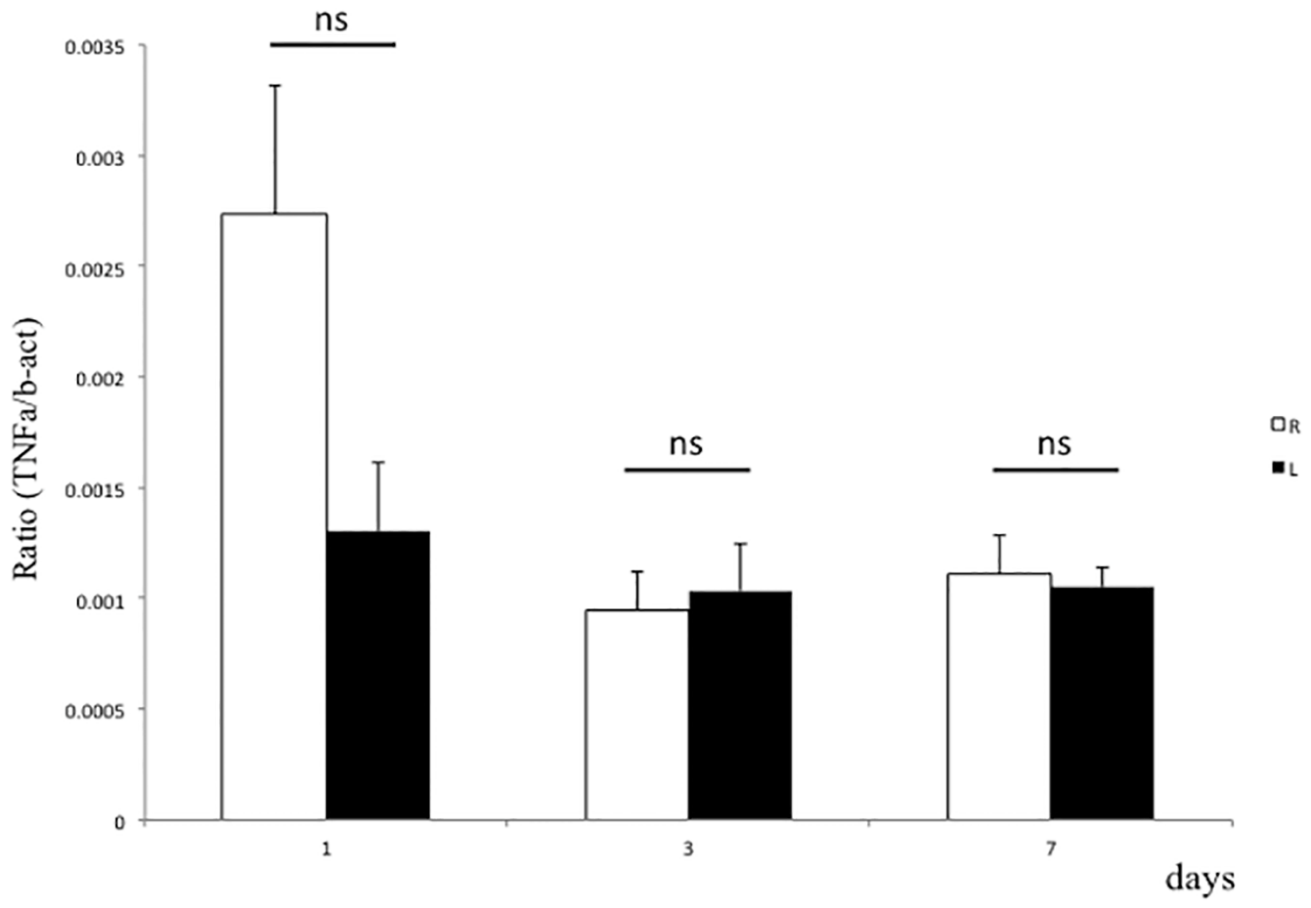
TNFα expression is not elevated in gastrocnemius muscle in a CRPS model. L5 spinal nerve ligation was performed on the left side of rats, and *TNFα* expression normalized to that of *β-actin* in gastrocnemius muscle was analyzed by realtime PCR at indicated time points. There was no significant difference in *TNFα* expression between ligated and non-ligated limbs. *p<0.05; **p<0.01; ns, not significant; n = 6; error bars = SEM.

**Fig 6:**
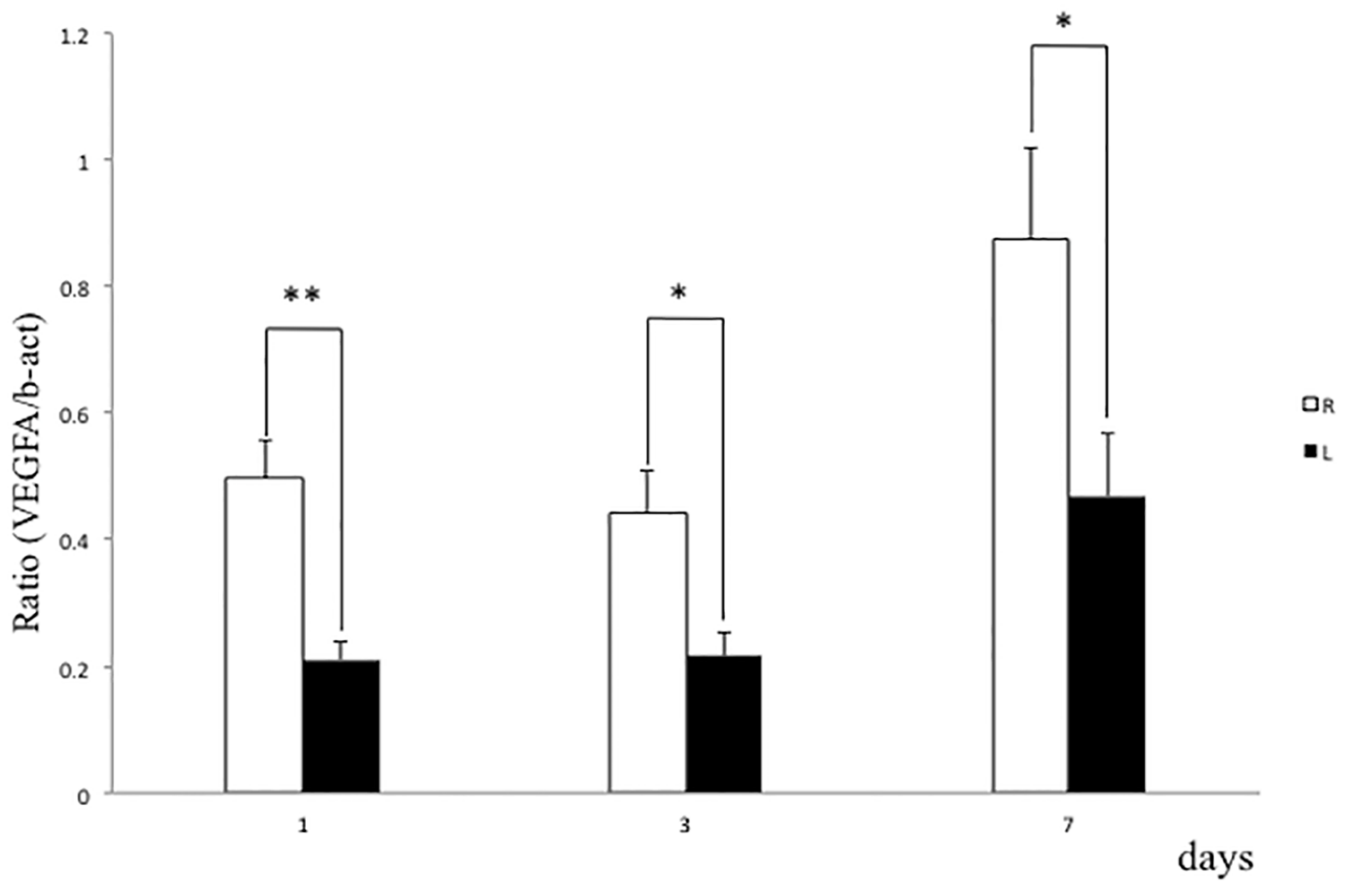
*VEGF* expression is down-regulated in gastrocnemius muscle by spinal nerve ligation. L5 spinal nerve ligation was performed on the left side in rats, and *VEGF* expression normalized to that of *β-actin* in gastrocnemius muscle was analyzed by realtime PCR at indicated time points. VEGF expression in gastrocnemius of the ligated left limb was significantly lower than that in the non-ligated right limb at one, three and five days after surgery. *p<0.05; **p<0.01; ns, not significant; n = 6; error bars = SEM.

Local inflammation or blood vessel dysfunction promotes edematous changes in tissues [49]. *TNFα* expression in gastrocnemius muscle did not increase following spinal nerve ligation (Fig 5). By contrast, *VEGF* expression significantly decreased in gastrocnemius muscle after ligation relative to the non-ligated side at days 1, 3 and 7 (Fig 6).

## Discussion

Although edema is one of the most typical symptoms of CRPS, there has been no study that measured the degree of edema quantitatively as a means of diagnosing CRPS. This study is the first report to measure both mechanical allodynia and the degree of edema quantitatively and simultaneously. Non-invasive methods to measure edema include volumetric measures (plethysmometer [50], bioimpedance [51], MRI, CT and ultrasound [52]) and circumferential measures [53]; however, none of those is as sensitive and accurate as the diffusion MRI measurement we present in this study. Since diffusion MRI can quantitatively detect edematous changes in particular tissues and distinguish volume changes due to by edema from other causes [26, 28, 30, 31, 33], we feel our method could be applied to quantitative diagnosis and used for follow-up or monitoring of treatment efficacy in CRPS patients by detecting edema. Our study also suggests that edematous changes may be due to suppressed *VEGF* expression in muscles with edema.

The SNL model of neuropathic pain has been widely used to investigative mechanisms underlying neuropathic pain as well as to screen new analgesic drugs [54–60]. CRPS-like symptoms develop by tightly ligating one (L5) or two (L5 and L6) segmental spinal nerves in animals. These procedures induce several types of neuropathic pain, such as ongoing pain, heat hyperalgesia, and mechanical and cold allodynia, all seen in human CRPS patients [35, 36, 61, 62], although the mechanisms of those symptoms underlying the SNL model are not fully disclosed. Here, we also confirmed that animals in the SNL model showed behavioral signs of mechanical allodynia in the experimental limb based on the von Frey hair assessment test using a DPA one day after surgery as compared to the contralateral limb, and those signs persisted throughout the experimental period. By contrast, the control group showed no sign of mechanical allodynia. Those outcomes correspond well with trends reported in previous studies using the SNL model [35, 36].

The expansion of extracellular space increases both ADC and T2 values [25–28]. We concluded that edematous changes were detected in skeletal muscle of the SNL model. The early increase of ADC ratios might reflect the less restriction of water molecules, which might be caused by increase of cell membrane permeability [63, 64]. The subsequent increase of T2 ratios indicates the increased volume of free water content in the extracellular space [26, 28, 31]. We found that from one to five days after surgery it is more useful to monitor ADC than T2 values to detect edematous changes in the SNL model. However, from day seven after surgery, measuring T2 values appeared to be a better option, as T2 ratios remained significantly high compared to the control group, while ADC ratios normalized.

The posterior tibial nerve is the main nerve that innervates gastrocnemius muscle, and the L5 spinal nerve is a unique nerve that consists of the posterior tibial nerve. For gastrocnemius muscle, damage to the posterior tibial nerve results in more severe than does damage to the L5 spinal nerve, and higher T2 ratios seen in our previous model of the posterior tibial nerve transection rats might reflect the degree of nerve damage [45]. Nonetheless, ADC ratios might useful to detect nerve damage but not sensitive enough to assess the degree of that damage. Although in our analysis it took longer to detect nerve damage based on T2 ratios relative to ADC ratios, we conclude that T2 is a useful tool to assess the degree of nerve damage.

Proposed mechanisms underlying CRPS fall into two categories: peripheral or central nerve system (CNS) changes. Recently reported examples of the former include focal small-fiber axonal degeneration [65], the presence of autoantibodies binding to the surface of peripheral autonomic neurons [66], and up-regulation of IL-6 and TNF-alpha levels in blister fluid [67]. One recent study relevant to CNS changes reports reorganization of the somatosensory cortex [68] and atrophy of regional grey matter [69].

To date, changes in local cytokine levels in edematous regions have not been reported in CRPS animal models. In our rat CRPS model, *TNFα* expression was unchanged, suggesting that local inflammation does not trigger edematous changes in muscles following nerve ligation. By contrast, expression of *VEGF*, which was originally identified as a vascular permeability factor [70], was significantly down-regulated in muscles following nerve ligation. VEGF is reportedly required for angiogenesis and vessel patterning, as well as neurogenesis [71], and also functions in vascular homeostasis [21]. Moreover, administration of anti-VEGF antibody reportedly promotes painful sensory neuropathy [72], while VEGF administration stimulates functional recovery of injured nerves accompanied by pain reduction in animals [73]. VEGF treatment also reportedly improves diabetic neuropathic pain and sensory neuronal degeneration [74]. Taken together, proximal nerve ligation likely down-regulates distal VEGF expression, leading to local edematous changes in distal target muscles and neuropathic pain. Further studies are needed to define mechanisms underlying regulation of distal VEGF expression by proximal nerve ligation.

CRPS diagnosis is now based on clinical symptoms, and there is no definitive, objective test to diagnose the condition. CRPS is a multifactorial disease, and objective tests are currently required to improve diagnostic accuracy. There have been several studies of the utility of MRI for this purpose. Those involve ^31^P nuclear MR Spectroscopy to reveal potential impairment of high energy phosphate metabolism in lower leg skeletal muscle [75], MRI using contrast materials to assess changes in skin thickness and tissue enhancement [76], and T2WI gadolinium dimeglumine enhancement on T1WI to detect hyperintense muscle signals which reverted to near normal following clinical improvement [77]. However, as yet no investigations have applied diffusion MRI to evaluate CRPS in patients. Here, we show that this methodology could detect early edematous changes in skeletal muscle using quantitative analysis of rat neuropathic pain models. The confirmation with a larger sample might clarify the results of the current study and that is our subject of the future study. We used a 1.5T clinical scanner to extend our technique for clinical use in the patient with CRPS. In order to compensate for the low SNR, we used thick slices (columns). On a 1.5T scanner, this is considered to be a reasonable compromise. Our findings overall suggest that diffusion MRI could provide a quantitative objective test to detect edematous changes in CRPS and that decreased VEGF levels may play a role in that pathology.
